# A study on the effects of vitamin D supplementation on hematological parameters and serum 25-hydroxy vitamin D in healthy dogs

**DOI:** 10.1186/s12917-024-04080-1

**Published:** 2024-05-24

**Authors:** Reza Gholipour Shahraki, Tahoora Shomali, Mahnaz Taherianfard, Nooshin Derakhshandeh, Saeed Nazifi, Ebrahim Abhaji

**Affiliations:** 1 Department of Basic Sciences, School of Veterinary Medicine, Shiraz University, P.O. Box 71441-69155, Shiraz, Iran; 2 Department of Clinical Sciences, School of Veterinary Medicine, Shiraz University, Shiraz, Iran

**Keywords:** Dog, Supplement, 25-hydroxy vitamin D3, Vitamin D3

## Abstract

**Background:**

Limited studies are available on vitamin D supplementation in dogs. This study evaluates the effect of a commercial vitamin D3 supplement on serum 25-hydroxy vitamin D as well as selected biochemical and hematological parameters in healthy dogs. Eight intact male adult dogs with a mean body weight of 20 kg from mixed breeds were included in the study. After adaptation period, dogs received vitamin D3 supplement at the dose of 50 IU/kg body weight per day. Blood samples were collected on days 0, 14, 28 and 42 of supplementation. Food was used for analysis of vitamin D3 content.

**Results:**

Significant increase in serum level of 25-hydroxy vitamin D3 was detected since day 14 of supplementation. Changes in serum 25-hydroxy vitamin D3 concentration during time showed an upward significance (*p* < 0.05). Vitamin D3 content of the food was 2900 IU/kg dry matter. Changes in serum phosphorus levels were upward significant. No dog showed calcium or phosphorus levels above the highest reference level. Liver and kidney parameters remained in the reference range during the experiment. A gradual significant increase was observed in hemoglobin and hematocrit which was started from day 14. Vitamin D3 supplementation had no significant effect on neutrophils, monocytes and lymphocytes percent during the study.

**Conclusions:**

Vitamin D3 supplementation at 50 IU/kg BW daily, increases serum levels of 25-hydroxy vitamin D in healthy dogs fed with a diet containing proper amount of this vitamin. It also increases hemoglobin and hematocrit levels in a time dependent manner without inducing adverse effects.

## Background

A century ago, the role of vitamin D in the health of bones and treatment of rickets was first described by McCollum and colleagues [1]. Today, it has been established that vitamin D receptors are widely expressed by nucleated cells and diverse health effects not only in bones but also in numerous other organs are described for this vitamin [2].

Synthesis of vitamin D3 in skin is started by the action of UVB radiation on 7-dehydrocholestrol which results in the production of pre-vitamin D3. Due to the high activity of the enzyme 7-dehydrocholesterol-reductase which converts 7-dehydrocholestrol into cholesterol, dogs have low ability to synthesize vitamin D in the skin and need to acquire this vitamin from dietary sources particularly fat, liver, blood and meat [3]. Therefore, dietary acquisition of vitamin D is crucial to prevent deficiency. On the other hand, many disease states which are not related to bones are shown to be associated with low vitamin D levels both in humans and dogs. Dogs with mastocytoma, protein-losing enteropathy, congestive heart failure, acute pancreatitis, neoplastic and non-neoplastic spirocercosis, blastomycosis, chronic renal failure etc. have shown lower serum concentrations of 25-hydroxy vitamin D3 as compared to healthy dogs [4]. However, it is not still clarified whether the reduction in serum 25-hydroxy vitamin D3 has a causal relationship with development of the disease or is just a consequence of the disease status [4]. Vitamin D supplementation seems quite important, both in order to prevent the deficiency in dogs that are fed with unstandardized diets and possibly to fight the diseases as mentioned above. It should be noted that excessive vitamin D consumption may result in hypercalcemia and vitamin D toxicosis in dogs [5]. Therefore, determination of the vitamin D dose that can be safely used in dogs with proper effect on the concentration of 25-hydroxy vitamin D as the most routinely assayed marker for this vitamin status is important.

Limited studies are available on the effects of vitamin D supplementation on circulatory 25-hydroxy vitamin D level in dogs and these studies are mostly focused on vitamin D administration in diet. In a sophisticated study by [6], six dogs were fed by a low vitamin D diet and received a treat with vitamin D2 (17.6 IU/kg) plus 1 of 3 doses of 25-hydroxy vitamin D3 (0, 12.2, or 24.5 IU/kg) once daily for 8 weeks. In the second trial by these authors, seven dogs were fed with a diet supplemented with vitamin D3 or 25-hydroxy vitamin D3 (3, 250 IU/kg and 16 µg/kg, respectively, as fed) for 10 weeks. The authors used HPLC method to assay 25-hydroxy vitamin D3 and 25-hydroxy vitamin D2 as well as 24, 25-dihydroxy vitamin D3 and 24, 25-dihydroxy vitamin D2. The study showed that dietary 25-hydroxy vitamin D3 was more potent than vitamin D3 to increase circulating 25-hydroxy vitamin D3 as the indicator of vitamin D status. No adverse effects were observed in studied dogs [6].

In contrast to cats, dogs have the ability to use both forms of vitamin D including vitamin D2 (ergocalciferol) and vitamin D3 (cholecalciferol) and can convert ergocalciferol to 25-hydroxy vitamin D in liver [4]. Recently, Jewell and Panickar evaluated the effect of feeding a diet containing vitamin D at 796, 3087, 5511, 7314, and 9992 IU/kg of dry matter for 6 months to adult dogs [7]. The three last doses were beyond the National Research Council (NRC) recommendation (552–3200 IU/kg dry matter) in the diet of adult dogs [8]. The authors found that dietary vitamin D supplementation was not associated with adverse effects on liver or kidney function parameters or serum calcium and phosphorus levels. Circulating 25-hydroxy vitamin D increased in all treated groups except in those that were fed with the lowest level of vitamin D [796 IU/kg] in diet [7].

In both of the above mentioned studies [6 and 7], a source of vitamin D was added to the diet. For dog owners, it may not be practical to prepare a well-mixed supplemented diet with the exact amount of added vitamin D. Additionally, when supplements are added to the diet, it is not always easy to calculate the precise dose an animal will receive based on its weight.

Vitamin D has a regulatory role in hematopoietic cells differentiation and proliferation [9]. It has been shown that 25-hydroxy vitamin D levels are correlated with red blood cell parameters in humans [10] and vitamin D deficiency is associated with a higher need for blood transfusions in human patients with sickle cell anemia [11]. Alizadeh and colleagues, 2022, investigated the relationship between 25-hydroxy vitamin D levels, hematology and serum biochemistry parameters in healthy dogs. These authors reported that serum 25-hydroxy vitamin D concentration was positively correlated with MCH value and the number of band neutrophils. They also described an inverse correlation between serum 25-hydroxy vitamin D levels and the number of blood eosinophils [12].

Considering limited available knowledge on vitamin D supplementation in dogs, this study tests the hypothesis that daily administration of a commercially available vitamin D supplement at a dose of 50 IU (1. 25 µg)/kg body weight for 6 weeks to healthy adult dogs is associated with increased serum 25-hydroxy vitamin D3 levels as the main indicator of vitamin D status. The possible effects of vitamin D supplementation on selected hematological parameters are also evaluated. Liver and kidney function tests and measurements of serum calcium and phosphorus levels are also performed to examine plausible adverse effects.

## Materials and methods

### Animals and study design

All procedures used in this study were approved by the institutional ethical committee and were compatible with Directive 2010/63/EU on the protection of animals used for scientific purposes.

Eight intact male adult (2–3 years old) dogs from mixed breeds were included in the study. Sample size was calculated based on the formula suggested by [13]. The dogs were owned by the Shiraz University, School of Veterinary Medicine. Mean body weight of dogs was 20 kg (in a range of 19–22 kg) with a body condition score of 3–4 on the 9-point scale. Dogs were clinically healthy and spent 2 weeks of adaptation period before starting the experiment. Blood samples were collected from all dogs for initial evaluation of routine liver and kidney parameters as well as complete blood count (CBC) with differential to exclude possibly sick animals. Dogs were kept in separate pens and were fed with 300 g/20 kg body weight (BW) dry dog food once a day as suggested by the manufacturer (Adult Nutripet dry dog food for dogs with moderate physical activity, Behintash Co., Tehran, Iran) and tap water *ad libitum*. According to the label, the food contained 21% crude protein, 9% fat, 3% fiber and 10% multivitamins and minerals. Dogs were checked twice a day for clinical signs. No clinical sign was detected during the study. In almost all visits no food left over was remained. Animals were taken for a walk for about 45 min/day. Dogs were weighed every week and no appreciable change was observed in their body weight during the study.

After adaptation period, all 8 dogs received vitamin D supplement in a commercial form (D-Vigel 1000®, Daana Pharma Co., Iran) at the dose of 50 IU/kg BW per day. The supplement was fed with food and was placed in a little treat. Venous blood samples were collected from the jugular vein on days 0, 14, 28 and 42 of the experiment at the maximum volume of 5 mL from each dog. Sampling was performed by using plain and EDTA vacutainer tubes for determination of serum biochemical parameters and hematological assays, respectively.

### Determination of hematological and biochemical parameters

Blood samples were taken to measure haematocrit (HCT), hemoglobin level (Hb), red blood cells (RBC) count, mean corpuscular hemoglobin (MCH), mean corpuscular volume (MCV) and mean corpuscular hemoglobin concentration (MCHC) and white blood cell (WBC) count with a veterinary hematology analyzer (Nihon Kohden, MEK-6450 Celltac Alpha, Tokyo, Japan). To determine the differential leukocyte counts (neutrophil, lymphocyte and monocyte), a drop of blood was thinly spread over a glass slide, air-dried, and stained with the Giemsa staining technique. One hundred cells were counted and classified.

Sera were harvested and kept at -20 °C until analysis. Serum biochemical parameters including creatinine, urea, calcium and phosphorus concentrations as well as alanine transaminase (ALT), aspartate transaminase (AST) and alkaline phosphatase (ALP) activities were measured with commercial kits (Biorex Fars, Shiraz, Iran) and analyzed using a biochemical autoanalyzer (Alpha Classic AT++, Sanjesh Company, Iran).

The reference range for almost all biochemical parameters are borrowed from [14]. Only for AST an in house reference was used.

### Determination of serum 25-hydroxy vitamin D levels and food vitamin D content

Serum 25-hydroxy vitamin D was measured using a solid-phase sandwich ELISA method (Biorex Fars, Shiraz, Iran) as described by the manufacturer.

A sample of food was used for analysis of vitamin D3 content as described previously by AOAC, 2005 [15].

### Statistical analysis

Data collected at each sampling time point was presented as mean ± SD. To evaluate the effect of vitamin D supplementation at different time points on each parameter repeated measures ANOVA was used followed by Tukey’s multiple comparison test. For calcium levels and monocyte percent Friedman test was used followed by Dunn’s multiple comparisons test. *p* < 0.05 was considered as the level of significance.

## Results

### Serum concentration of 25-hydroxy vitamin D3

**Fig. 1:**
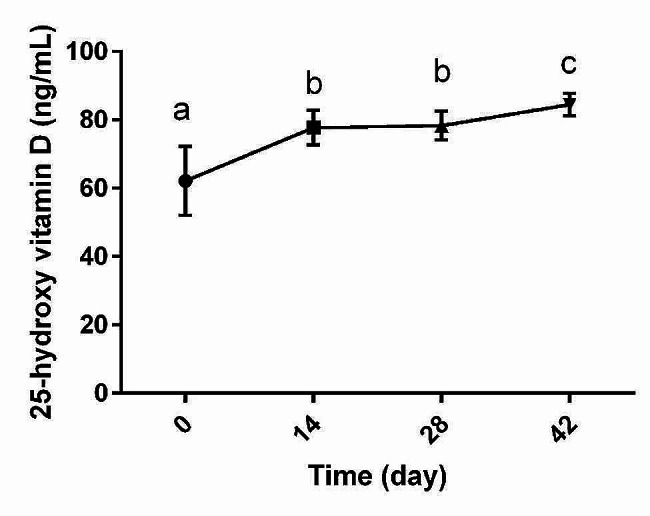
Changes in serum concentrations of 25-hydroxy vitamin D3 (mean and SD) during time in dogs (*n* = 8) supplemented with vitamin D administered daily at 50 IU (1.25 µg) per kg body weight. Different letters are used to show significant difference among different time points (*p* < 0.05)

On day 0, mean serum concentration of 25-hydroxy vitamin D3 of dogs was 62.1 ng/mL. After 42 days of vitamin D supplementation, this parameter’s value reached to 84.5 ng/mL that shows an increase of about 35%. Changes in 25-hydroxy vitamin D3 concentration due to supplementation of vitamin D showed an upward significance (*p* < 0.0001) during the whole time of the study. Significant increase in serum level of 25-hydroxy vitamin D3 was detected since day 14 of supplementation (*p* < 0.05 vs. day 0). While 25-hydroxy vitamin D3 concentration was statistically the same between day 14 and day 28 (*p* > 0.05), a significant increase was observed in this parameter on day 42 compared to 28 (*p* < 0.05). Changes in serum level of 25-hydroxy vitamin D3 during time is summarized in Fig. 1.

### Vitamin D3 content of the food

Vitamin D3 content of the food was determined to be 72.5 µg/kg or 2900 IU/kg dry matter. The National Research Council (NRC) recommends 552–3200 IU vitamin D3/kg food dry matter for maintenance of adult dogs [9]. Therefore, the food which was used in the present study contained proper amounts of vitamin D3.

### Serum calcium and phosphorus levels

Results related to serum calcium and phosphorus levels are summarized in Table 1. Vitamin D3 supplementation was associated with a gradual but insignificant (*p* > 0.05) increase in serum calcium levels of dogs during the time of experiment. The highest calcium level was observed on day 42, which was not significantly different from the baseline (day 0) (*p* > 0.05). Changes in serum phosphorus levels were upward significant (*p* = 0.0001). Serum phosphorus levels on day 42 were significantly higher than baseline (*p* < 0.05) and day 14 (*p* < 0.01). Moreover, dogs had appreciably higher serum phosphorus levels on day 28 compared to day 14 (*p* < 0.05).

No dog showed calcium or phosphorus levels above the highest reference level (above 11.7 mg/dL and 5.3 mg/dL for calcium and phosphorus, respectively [14]).

**Table 1:** Serum total calcium and phosphorus levels (mean ± SD) of dogs (*n* = 8) in different sampling time points after supplementation with vitamin D. The presence of at least one common superscript letter shows that values in a row are not significantly different (*p* > 0.05) and vice versa

	Day 0	Day 14	Day 28	Day 42
Calcium (mg/dL)	8.40 ± 0.622^a^	8.63 ± 0.892^a^	8.71 ± 0.451^a^	9.42 ± 0.566^a^
Phosphorus (mg/dL)	3.18 ± 0.432^ab^	2.84 ± 0.354^a^	3.91 ± 0.445^bc^	4.40 ± 0.589^c^

### Liver and kidney parameters

**Fig. 2:**
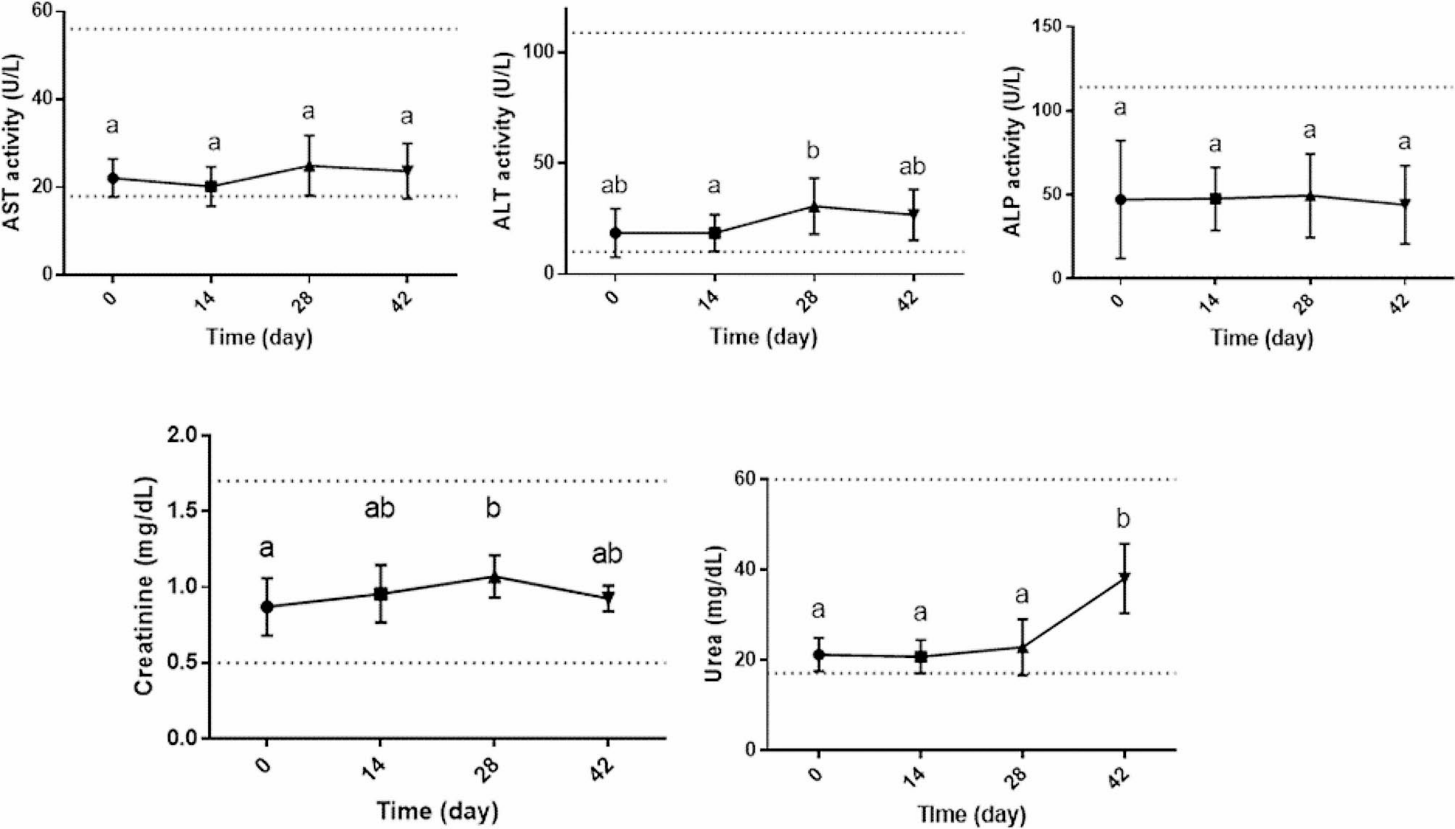
Liver and kidney function parameters (mean and SD) of dogs (*n* = 8) in different sampling time points after supplementation with vitamin D. The presence of at least one common superscript letter shows that values related to a parameter in different time points are not significantly different (*p* > 0.05) and vice versa. Dotted lines indicate the reference range

Although some fluctuations were observed in liver and kidney function parameters of dogs during the experiment, vitamin D supplementation was not associated with clinically important changes of these parameters. All of the parameters remained in the reference range during the experiment (Fig. 2).

### Hematological parameters

Table 2 summarizes changes in blood cell parameters of dogs supplemented with vitamin D at different sampling time points.

In the present study, vitamin D supplementation was not associated with a significant change in RBC count, MCV, MCH and MCHC of dogs after 14, 28 or 42 days of supplementation compared to day 0 (*p* > 0.05). However, a gradual increase was observed in hemoglobin and hematocrit which was started from day 14. The effect of treatment during time was significant for both of these parameters (*p* = 0.0022 for hemoglobin and *p* = 0.0096 for hematocrit). On day 42 of vitamin D supplementation dogs had significantly higher hemoglobin and hematocrit as compared to day 0 (*p* < 0.01 and *p* < 0.05, respectively).

Regarding WBCs, vitamin D supplementation had no significant effect on WBC count, neutrophils, monocytes and lymphocytes percent during the study (*p* > 0.05).

**Table 2:** Hematological parameters (mean ± SD) of dogs (*n* = 8) in different sampling time points after supplementation with vitamin D. The presence of at least one common superscript letter shows that values in a row are not significantly different (*p* > 0.05) and *vice versa*

	Day 0	Day 14	Day 28	Day 42
RBC number (×10^6^/mL)	6.10 ± 0.911^a^	6.40 ± 0.526^a^	6.85 ± 0.627^a^	6.94 ± 0.611^a^
Hemoglobin (g/dL)	12.8 ± 1.32^a^	13.9 ± 0.817^a^	14.6 ± 1.17^ab^	15.2 ± 1.13^b^
Hematocrit (%)	38.2 ± 4.33^a^	41.3 ± 2.31^ab^	43.7 ± 3.55^ab^	44.3 ± 3.41^b^
MCV (fL)	63.0 ± 2.93^a^	64.7 ± 3.17^a^	63.9 ± 2.23^a^	63.9 ± 2.31^a^
MCH (pg)	21.1 ± 1.47^a^	21.7 ± 1.09^a^	21.4 ± 0.910^a^	21.9 ± 0.895^a^
MCHC (g/dL)	33.5 ± 0.789^ab^	33.5 ± 0.361^ab^	33.4 ± 0.328^a^	34.2 ± 0.584^b^
WBC number (×10^3^/µL)	11.6 ± 2.02^a^	11.7 ± 2.32^a^	11.4 ± 1.63^a^	12.5 ± 1.63^a^
Neutrophils (%)	68.2 ± 11.9^a^	67.1 ± 7.18^a^	66.5 ± 12.2^a^	66.1 ± 10.7^a^
Lymphocytes (%)	30.7 ± 11.6^a^	32.5 ± 7.44^a^	31.3 ± 12.6^a^	33.7 ± 10.7^a^
Monocytes (%)	1.00 ± 1.15^a^	0.285 ± 0.488^a^	0.857 ± 0.378^a^	0 ± 0^a^

RBC: red blood cells; MCV: mean corpuscular volume; MCH: mean corpuscular hemoglobin; MCHC: mean corpuscular hemoglobin concentration and WBC: white blood cell.

## Discussion

In the present study we observed that vitamin D3 supplementation at 50 IU/kg BW daily for 6 weeks, increases serum levels of 25-hydroxy vitamin D in healthy dogs fed with a diet containing proper amount of this vitamin. It also increases hemoglobin and hematocrit levels in a time dependent manner without inducing adverse effects on serum liver or kidney function parameters or serum calcium and phosphorus concentrations.

In most of the previous studies [6, 7 and 16], vitamin D supplementation to dogs was accomplished by adding the vitamin D to diet. This method has some disadvantages including difficulties in preparing a well mixed-up supplemented diet by the owner or precise calculation of the supplement dose received by the animal per kg body weight.

The only available study that has used a formulation of vitamin D3 as the source for vitamin D3 supplementation is performed by [17]. In this study, 7 dogs were supplemented with a relatively high dose vitamin D3 (122 IU/kg BW, daily) with food which is five times the recommended allowance but within the limit recommended by NRC for maintenance of adult dogs. The vitamin D3 was formulated with olive oil and vitamin E (to prevent oxidation of the oil) by the investigators and was administered to the dogs for 9–10 weeks. These researchers reported that administration of the supplement was associated with a slow increase in serum concentration of 25-hydroxy vitamin D which was significantly higher than control group only after 9–10 weeks of supplementation.

On the contrary, in the present study we observed that supplementation of dogs with 50 IU/kg BW vitamin D, about 0.4 of the dose which was used by [17] (122 IU/kg BW), resulted in increased serum 25-hydroxy vitamin D that was observed from day 14 after initiation of supplement administration and reached the highest level after 6 weeks. The reason for this discrepancy may be the fact that in the study performed by [17], the daily dose was administered by dog owners. Although the authors declare that owners were instructed not to alter diet, environment and exercise schedule during the experiment; the degree of commitment of the owners to administer the supplement is only assessed by weighing the supplement at the beginning and end of the trial. In the present study administration of the supplement was performed by a trained person and the accuracy of the procedure was monitored every day.

The inclusion of a parallel control group is one of the strength points of the study performed [17]. However, vitamin D intake from the diet consumed by supplemented dogs was significantly lower than control dogs which were included in this study. Since dogs acquire vitamin D only from dietary sources, vitamin D content of the diet is a determining factor. Although these authors declare that no significant difference was found in serum 25-hydroxy vitamin D within supplemented dogs group during the experiment, a gradual increase can be observed from week 3 post supplementation.

The median value of serum 25-hydroxy vitamin D of dogs in the beginning of our study was 59.9 ng/mL. Consistently, in a study performed by [12], median serum concentration of 25-hydroxy vitamin D of 90 healthy dogs showed a median of 52.5 ng/mL by ELISA method.

In agreement with previous report related to vitamin D supplementation in dogs [7], in the present study, vitamin D supplementation was not associated with adverse effects on kidney and liver function parameters of the studied dogs nor a drastic derangement in serum calcium or phosphorus levels.

A considerable amount of knowledge is available on the relationship between vitamin D deficiency and anemia and also the effect of vitamin D supplementation on hemoglobin and other red blood cell parameters in humans [18–20], on the contrary, data on this subject is scarce in veterinary species.

In a study by [12]; a direct correlation was observed between serum levels of 25-hydroxy vitamin D and MCH in healthy dogs. These authors did not find any correlation between serum 25-hydroxy vitamin D and lymphocytes, monocytes and neutrophils count of the dogs. In another study by [21] hospitalized cats with neutrophilia had lower serum 25-hydroxy vitamin D concentrations than cats with neutrophil concentrations below the upper limit of the reference interval. A single bolus injection of vitamin D3 has been associated with increased hemoglobin and hematocrit and decreased lymphocyte and granulocyte count of healthy Holstein bulls [22].

In a study by [16], administration of a diet supplemented with 16 µg/kg of 25-hydroxy vitamin D3 to dogs with vitamin D insufficiency for 4 months was associated with a significant increase in hemoglobin, hematocrit and erythrocyte number while the dogs that received diets supplemented with 3240 IU/kg vitamin D3 for the same period showed only increased values of MCV. As previously stated, we observed that supplementing dogs with 50 IU/kg BW is associated with increased hemoglobin and hematocrit of dogs after 6 weeks. It seems that dosage regimen, type of the vitamin D supplement and route of administration (supplemented diet vs. a dietary supplement form) among other factors can affect the response to vitamin D supplementation. The effect of vitamin D supplementation on red blood cell parameters in anemic dogs remains to be clarified in future studies.

In our study, 6 weeks of vitamin D supplementation was not associated with a change in WBC count, neutrophils, lymphocytes and monocytes percent. Consistently, in a study on human subjects, Wall-Gremstrup and colleagues [23] reported that high dose vitamin D supplementation has no beneficial effect on WBC count of men with infertility. Similar results are reported by [16] in dogs supplemented with 25-hydroxy vitamin D3 or vitamin D in diet.

Serum PTH assay may help in acquiring knowledge on the possible effect of vitamin D supplementation on this hormone’s level. In a study by [6], dietary supplementation of dogs with 25-hydroxy vitamin D3 for 8 weeks was not associated with a significant change in serum PTH levels despite an appreciable increase in serum 25-hydroxy vitamin D concentration. It has been demonstrated that the lowest concentration of 25-hydroxy vitamin D in dogs which is necessary to suppress PTH synthesis is 100–120 ng/mL [24]. In the study performed by [6], the median level of 25-hydroxy vitamin D at the end of the study was 67.2 ng/mL where the median 25-hydroxy vitamin D in serum of dogs in our study reached to 83.2 ng/mL after 6 week of supplementation, both of the values are well below the concentrations needed to suppress PTH.

In conclusion, vitamin D supplementation for 6 weeks at a dose of 50 IU/kg BW daily provided by a commercial product increases serum levels of 25-hydroxy vitamin D and also hemoglobin and hematocrit in a time dependent manner in healthy dogs without inducing adverse effects.

## Data Availability

The data that support the findings of this study are not openly available due to reasons of sensitivity and are available from the corresponding author upon reasonable request.
